# Dataset of barcoded Reticulariaceae: ten years of DNA sequencing

**DOI:** 10.3897/BDJ.12.e115630

**Published:** 2024-02-28

**Authors:** Dmytro Leontyev, Iryna Yatsiuk

**Affiliations:** 1 Department of Botany, H.S. Skovoroda Kharkiv National Pedagogical University, Kharkiv, Ukraine Department of Botany, H.S. Skovoroda Kharkiv National Pedagogical University Kharkiv Ukraine; 2 Institute of Ecology & Earth Sciences, University of Tartu, Tartu, Estonia Institute of Ecology & Earth Sciences, University of Tartu Tartu Estonia

**Keywords:** biodiversity, Cribrariaceae, database, Eumycetozoa, GBIF, geographic coordinates, Lucisporomycetidae, Myxogastrea, Myxomycetes, species distribution

## Abstract

**Background:**

As a result of the ten years (2012–2022) work under the critical revision of the genera of Reticulariaceae, a set of papers was published. Collection data of hundreds of specimens, used as a material for these studies, were provided as supplements of corresponding papers, but remained unpublished in biodiversity databases.

**New information:**

Here, we represent an occurrence dataset "Barcoded Reticulariaceae of the World", published in GBIF. It includes data on 523 myxomycete collections (including 36 types) gathered from five continents and spanning 24 countries. The dataset encompasses 43 distinct species and one subspecies of myxomycetes, including rare, endemic, and recently-described taxa. Species included to the database mainly belong to the genera *Alwisia*, *Lycogala*, *Reticularia*, *Siphoptychium*, *Thecotubifera* and *Tubifera* (Reticulariaceae), but as well *Lindbladia* and *Licaethalium* (Cribrariaceae). Nearly all of the research material, with the exception of several old collections, underwent molecular barcoding, primarily involving the 18S rDNA gene, but also the elongation factor 1α gene and mitochondrial cytochrome oxidase subunit I gene. For those sequences that are stored in the NCBI GenBank, accession numbers are provided in the dataset. Newly-described species make up a significant part of the studied herbarium collections; many of them can be characterised as common for their region. A particularly high level of taxonomic novelty is observed in Australia, which may be explained by the endemism of the local myxomycete biota.

## Introduction

Reticulariaceae is a well-known family of myxomycetes, the first representatives of which were described in the 17^th^-18^th^ centuries (Leontyev et al. 2022b,Leonytev and Schnittler 2022). In 2012, the family contained three genera and 25 accepted species (Lado 2005), including the trivial *Lycogalaepidendrum* (L.) Fr. and *Tubiferaferruginosa* (Batsch) J.F. Gmel. In 2012, Dmytro Leontyev (co-author) initiated a critical revision of the family, which was carried out during 2012–2023 through several projects (See Projects section). These studies increased the number of species of the Reticulariaceae to 52 (Lado 2005), the number of genera to six and one more genus, *Licaethalium*, was re-erected within the Cribrariaceae for species, previously classified within Reticulariaceae (Leontyev et al. 2019b). Fifteen species of the genus *Lycogala*, nine species and one subspecies of the genus *Tubifera*, three species of the genus *Alwisia* and two species of the genus *Siphoptychium* were described (Leontyev et al. 2014a, Leontyev et al. 2014b,Leontyev et al. 2015, Leontyev et al. 2019a, Lloyd et al. 2019, Leontyev et al. 2022a, Leontyev et al. 2022b, Leontyev and Schnittler 2023b, Leontyev et al. 2023). The classical species *L.epidendrum* and *T.ferruginosa* have proven to represent species complexes, which contain at least 13 and 76 biological species, respectively (Leontyev and Schnittler 2023a).

The critical re-evaluation of Reticulariaceae involved an integrative approach that combined a comprehensive morphological assessment with molecular barcoding of specimens. The molecular study, aimed primarily at identifying operational taxonomic units, but also at tracing phylogenetic relationships between them, covered almost the entire collection. In this regard, the material of the ten-year critical revision of Reticulariaceae represents the set of data unprecedented in terms of depth of study and accuracy of identification. Until now, these data had only been available as supplementary files in respective publications, making it inaccessible for broader analysis. In this paper, we present a dataset "Barcoded Reticulariaceae of the World" containing all this information, which we have now published on GBIF (Leontyev and Yatsiuk 2023) in compliance with FAIR principles (findable, accessible, interoperable, reusable). In the dataset, we provide data on 523 myxomycete records on 41 species and one subspecies of Reticulariaceae and two species of Cribrariaceae. Amongst them, 36 collections represent nomenclatural types. Nearly all (520, 99.4%) collections are accompanied with geographic coordinates; 446 (85.3%) of collections are barcoded by at least one marker gene, including 18S rDNA gene – 435 (83.2%), elongation factor 1α gene (EF1α) – 68 (12.9%) and the mitochondrial cytochrome oxidase subunit I gene (COI) – 58 (11.0%).

The primary goal of the database is to make data on the global distribution of Reticulariaceae species available for broader studies. Significant changes in the taxonomy of this family due to its critical revision have not yet been incorporated into identification literature. Species described during last several years are not yet represented in the GBIF or they are represented only by data, provided by non-institutional research via iNaturalist and, thus, not supported by molecular data or microscopy. Another problem is that many ‘classical’ species, such as *Tubiferaferruginosa*, *T.casparyi* (Rostaf.) T. Macbr., *T.microsperma* (Berk. & M.A. Curtis) G.W. Martin, *Lycogalaepidendrum*, and *L.exiguum* Morgan have been redefined more narrowly, based on molecular data. Consequently, their geographical distribution appeared to be more limited than previously believed and available data on the distribution of these taxa should, therefore, be used with caution. Our dataset offers reliable information about the distribution of both recently-described and revised species, providing data, confirmed through molecular barcoding and meticulous morphological studies.

In our barcoding project, we thoroughly examined all the herbarium specimens available in various collections (these were around 30 herbaria and private collections from Germany, US, Costa Rica, Australia and other counties, listed in publications, cited above). Consequently, the distribution of species, based on the number of specimens, can offer a somewhat biased, yet valuable indication of their global prevalence. The species with the highest abundance (see the Taxonomic coverage secion) are *L.epidendrum* and *T.ferruginosa*. This should not come as a surprise since both species were described in the 18^th^ century and are found worldwide. However, these species are followed in the abundance list by *Siphoptychiumreticulatum* Leontyev, Schnittler & S.L. Stephenson, *T.vanderheuliae* S.J. Lloyd, Leontyev & Dagamac, *L.leopardinum* Leontyev, Ishchenko, Schnittler & E. Johannesen and *T.montana* Leontyev, Schnittler & S.L. Stephenson. All of these taxa are relatively recent discoveries, all made within the scope of our ten-year project (Leontyev et al. 2015, Leontyev et al. 2019a, Leontyev et al. 2023). It is reasonable to assume that species previously unknown to science before our studies are not necessarily rare. The 'invisibility' of these species to taxonomists before the start of our project can be attributed solely to the limitations of the morphological criteria that were initially used to distinguish taxa within Reticulariaceae. This conjecture is supported by the fact that much rarer species, such as *S.casparyi* and *Licaethaliumolivaceum* (Ehrenb.) Rostaf., which exhibit distinct morphological characteristics, were described as early as the 19^th^ century (Lado 2005, Leontyev et al. 2019b). By adopting an integrative approach, at the core of which was the quest for morphological features correlated with molecular barcoding data, we were able to delimit species previously unknown to science that are widespread in extensively studied regions like Europe and North America (Leontyev et al. 2014b, Leontyev et al. 2015, Leontyev et al. 2023).

The percentage of newly-discovered species in herbarium material obtained from various continents is an intriguing aspect. Australia stands out as the leader in this regard, with a remarkable 88% of the Reticulariaceae specimens collected in the country turning out to be new species. Nevertheless, Europe and North America also present impressive results, with 52% and 51%, respectively. These values are probably biased, primarily because we did not include a proportionate number of specimens from *L.flavofuscum* (Ehrenb.) Rostaf., *Reticularajurana* Meyl., *R.lycoperdon* Bull., and *R.splendens* Morgan in our studies. This omission occurred because these species exhibit clear morphological distinctions and were found to be taxonomically uniform, based on the sequencing of marker genes. Consequently, the abovementioned percentage of novelty within the studied collections is somewhat inflated. However, as a relative assessment, it can still offer valuable insights and this supposition gains support from the independent evidence of a unique myxomycete flora in Australia (Lloyd 2022).

## Project description

### Title

Barcoded Reticulariaceae of the World

### Personnel

Dmytro Leontyev, Iryna Yatsiuk

### Study area description

Worldwide

### Funding

The project if partially funded by individual research grants to the first author by: (1) German Academic Exchange Foundation (DAAD) programme, Greifswald, Germany, 2012; (2) Fulbright Program, Fayetteville, AR, USA, 2013–2014; (3) RESPONSE programme of the German Foundation for Fundamental Research (DFG), Greifswald, Germany, 2017 and (4) Alexander von Humboldt Foundation programme, Greifswald, Germany, 2019–2023. Local partners of the project were Prof. Martin Schnittler (for the programmes implemented in Germany) and Prof. Steven Stephenson (for the programme implemented in the USA).

## Sampling methods

### Sampling description

The material of the study encompasses collection data about myxomycete specimens, which were identified and barcoded by the authors of the publications within the framework of the project on critical revision of genera of Reticulariaceae in 2012–2022.

Material from the abovementioned studies was obtained by their authors both directly from fieldwork and as loans from numerous herbaria and individuals, with or without institutional affiliation (see acknowledgements in the cited paper and collector data in the dataset). The total number of collections analysed was over 1500, but only 523 were included in published studies and identified to the species level and are included in the present dataset.

Locality information was either obtained by the authors directly during field studies or recorded from herbarium labels. Coordinates, regardless of the original format, are presented as decimal fractions of a degree. Nearly all of the research material, with the exception of several old collections, underwent molecular barcoding, primarily involving the 18S rDNA gene, but also EF1α and COI. For those sequences that are stored in the NCBI GenBank, accession numbers are provided in the dataset. It should be noted that identical sequences obtained from different specimens were not published in GenBank as separate records in the 2014–2019 publications. Therefore, for most of the *Alwisia*, *Siphoptychium* and *Tubifera* occurrences in the dataset, the records with which they share an identical barcode are given, rather than their own GenBank accession numbers. In such cases, the marker “ident.” is added to the accession number, which means “barcode is identical to this accession number”.

### Quality control

Spreadsheets were checked and cleaned with Openrefine v. 3.2 (Ham 2013). Results of georeferencing were checked visually by plotting occurrences with GPSVisualizer (https://www.gpsvisualizer.com/map_input?form=data). Taxonomic assessment was based upon our own publications, but also verified with the database “An online nomenclatural information system of Eumycetozoa” (Lado 2005).

### Step description

The occurrence dataset was produced with the following steps:

1. Preparation of Darwin Core-formatted template;
2. Data extraction from the Excel sheets (mostly Supplementary files of the aforementioned publications) into corresponding columns;
3. Taxonomic assessment of names;
4. When necessary, manual georeferencing of occurrences. If the coordinates of the occurrence were missing in the literature, the occurrence was georeferenced based on text description of the occurrence location. Precision was determined according to the accuracy of the distance to the occurrence from the authors' description in the text. The accuracy of the given coordinates is determined as follows: one number in decimal place corresponds to the precision of 11.1 km, two numbers = 1.11 km, with each subsequent sign, the distance is reduced by a factor of 10. WGS84 was used as a spatial reference system.
5. Data cleaning using OpenRefine;
6. Matching species names;
7. Plotting of occurrences on map and visual checkup of coordinates.

## Geographic coverage

### Description

Worldwide land area.

**Figure 1.**
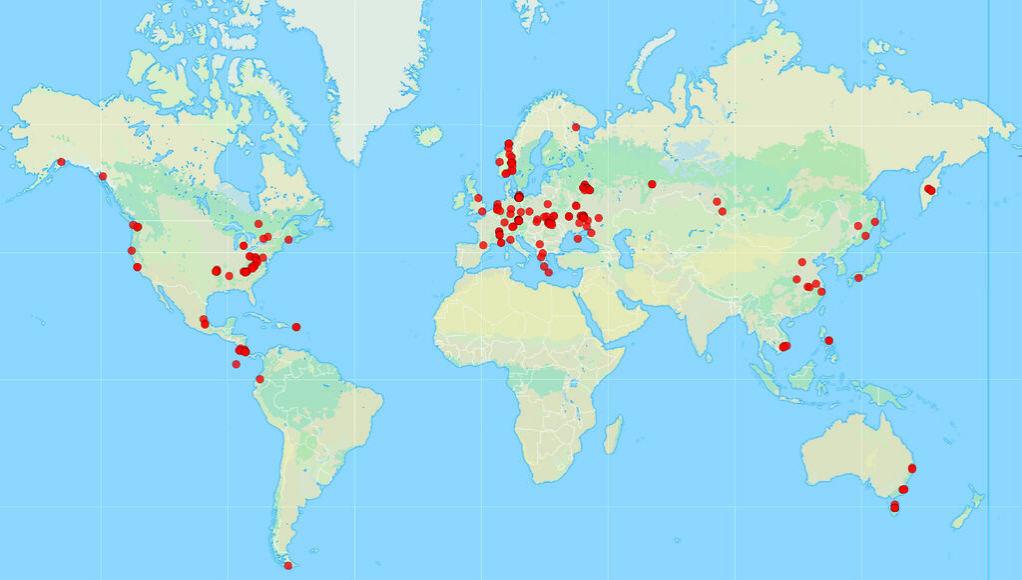
Collection sites of myxomycete specimens, included in the dataset "Barcoded Reticulariaceae of the World". The map was created using the on-line service GPSVisualizer (https://www.gpsvisualizer.com/map_input?form=data).

Material was gathered from five continents and represents 24 countries (Fig. 1). The distribution of occurrences in our dataset reflects, first of all, the invested research effort. Most of records originate from the Europe, US, Central America, Eastern China and Australia, which are known as areas well studied by myxomycetologists. In contrast, molecular signatures of Reticulariaceae remain understudied in Africa, South America, South Asia including the Indian subcontinent, the boreal regions of Asia and North America. Undoubtedly, investigations of specimens collected in these regions will contribute significantly to expanding our understanding of the diversity within this family.

## Taxonomic coverage

### Description

**Figure 2.**
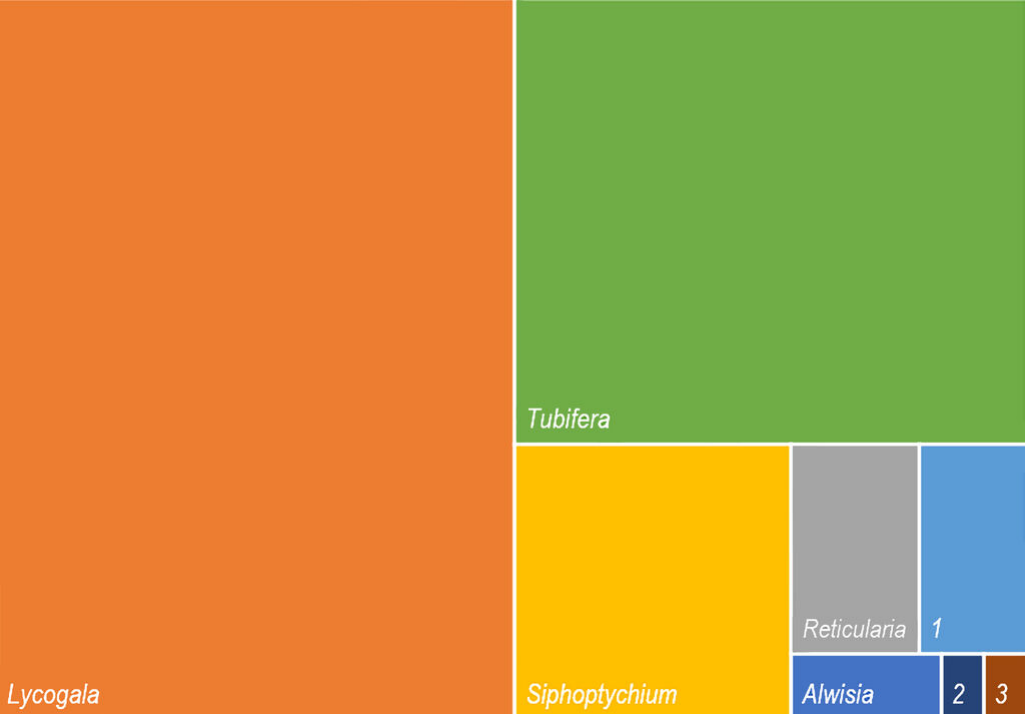
Relative number of occurrences in genera of Reticulariaceae and Cribrariaceae, represented in the dataset "Barcoded Reticulariaceae of the World".1 – *Thecotubifera*. 2 – *Licaethalium*. 3 – *Lindbladia*.

**Figure 3.**
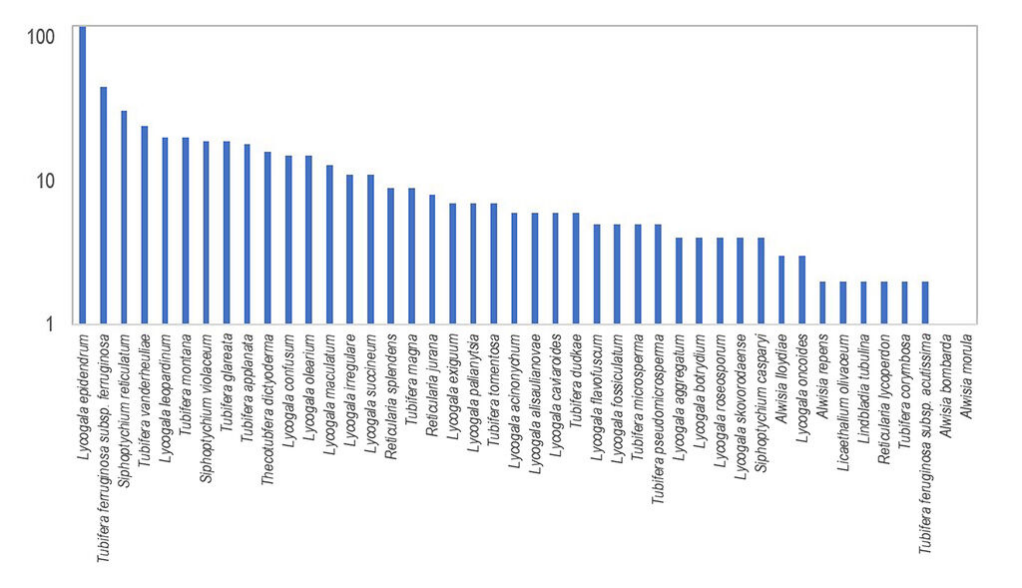
Number of occurrences per species of Reticulariaceae and Cribrariaceae, represented in the dataset "Barcoded Reticulariaceae of the World" (logarithmic scale).

The dataset includes data on 523 myxomycetes occurrences on the genera *Alwisia* (7), *Lycogala* (265), *Reticularia* (19), *Siphoptychium* (54), *Thecotubifera* (16) and *Tubifera* (158), but as well *Lindbladia* (2) and *Licaethalium* (2) from Cribrariaceae (Fig. 2). The most abundant species are *Lycogalaepidendrum*, *Tubiferaferruginosa*, *Siphoptychiumreticulatum*, *T.vanderheuliae*, *L.leopardinum* and *T.montana* (Fig. 3).

### Taxa included

**Table:** 

Rank	Scientific Name	
class	Myxomycetes	
family	Reticulariaceae	
family	Cribrariaceae	

## Temporal coverage

### Notes

Data range: 1893–2021

Amongst dated collections, 90% were made after 2000.

## Usage licence

### Usage licence

Open Data Commons Attribution License

## Data resources

### Data package title

Barcoded Reticulariaceae of the World

### Resource link

https://doi.org/10.15468/5xa74w

### Alternative identifiers

https://www.gbif.org/dataset/2b767e54-7921-4908-af93-84a9c35264c1

### Number of data sets

1

### Data set 1.

#### Data set name

Barcoded Reticulariaceae of the World

#### Data format

Darwin Core

#### Download URL

https://ukraine.ipt.gbif.no/archive.do?r=reticulariaceae

#### Description

The dataset includes a tabulation-delimited table with 25 fields in Darwin Core terms and 523 records in it (Leontyev and Yatsiuk 2023).

**Data set 1.** 

Column label	Column description
occurrenceID	https://dwc.tdwg.org/terms/#dwc:occurrenceID; the identifier for the occurrences, the universal unique identifier (UUID) was used for this purpose
basisOfRecord	http://rs.tdwg.org/dwc/terms/basisOfRecord; PreservedSpecimen for all occurrences, since all are herbarium specimens
catalogNumber	http://rs.tdwg.org/dwc/terms/catalogNumber; identifier of the specimen within the herbarium
otherCatalogNumbers	http://rs.tdwg.org/dwc/terms/otherCatalogNumbers, alternative identifier of the specimen; appears in cases where a part of the specimen was shared with another collection and a duplicate specimen was created
taxonRank	http://rs.tdwg.org/dwc/terms/taxonRank; the lowest taxonomic rank of the occurrence.
scientificName	http://rs.tdwg.org/dwc/terms/scientificName; The full name of the currently accepted taxon, name after the taxonomical assessment as described in Methods.
country	https://dwc.tdwg.org/terms/#dwc:country.
countryCode	https://dwc.tdwg.org/terms/#dwc:countryCode.
geodeticDatum	https://dwc.tdwg.org/terms/#dwciri:geodeticDatum; The geodetic datum upon which the geographic coordinates given in dwc:decimalLatitude and dwc:decimalLongitude are based, one value, WGS84.
decimalLatitude	http://rs.tdwg.org/dwc/terms/decimalLatitude; geographic latitude in decimal degrees.
decimalLongitude	https://dwc.tdwg.org/terms/#dwc:decimalLongitude; geographic longitude in decimal degrees.
coordinateUncertaintyInMetres	https://dwc.tdwg.org/terms/#dwc:coordinateUncertaintyInMeters; the distance (in metres) from the given decimalLatitude and decimalLongitude describing the smallest circle containing the whole of the Location. Set as described in Methods.
georeferencedBy	https://dwc.tdwg.org/terms/#dwc:georeferencedBy; name of the person who georeferenced the occurrence.
year	http://rs.tdwg.org/dwc/terms/year; year or a range of years in which the occurrence was recorded.
month	http://rs.tdwg.org/dwc/terms/month; month or a range of months in which the occurrence was recorded.
day	http://rs.tdwg.org/dwc/terms/day; day or a range of days of the month in which the occurrence was recorded.
eventDate	https://dwc.tdwg.org/terms/#dwc:eventDate; the full date of the observation as precisely as it could be extracted from the publication.
stateProvince	https://dwc.tdwg.org/terms/#dwc:stateProvince; the name of the next smaller administrative region than country (province, region etc.)
county	http://rs.tdwg.org/dwc/terms/county; The full, unabbreviated name of the next smaller administrative region than stateProvince (county, shire, department etc.)
municipality	https://dwc.tdwg.org/terms/#dwc:municipality; the full, unabbreviated name of the next smaller administrative region than county (city, municipality etc.)
locality	http://rs.tdwg.org/dwc/terms/locality; more specific description of the locality than municipality, derived from the original.
habitat	http://rs.tdwg.org/dwc/terms/habitat; description of macrohabitat such as vegetation type and microhabitat, such as a substrate type.
recordedBy	http://rs.tdwg.org/dwc/iri/recordedBy; the collector/s of the specimen.
associatedSequences	http://rs.tdwg.org/dwc/terms/associatedSequences; GenBank accession numbers for partial SSU, EF1α and COI genes separated with |. If the sequence has no individual GenBank accession number, the term “ident.” (identical to) is applied after the accession number of an identical sequence of another specimen.
typeStatus	http://rs.tdwg.org/dwc/terms/typeStatus; indicating whether an occurrence is a nomenclatural type for a corresponding scientificName.
